# Supplementary material for: A 2D chiral microcavity based on apparent circular dichroism
Source: Nat Commun. 2024 Apr 9;15:3072. doi: 10.1038/s41467-024-47411-4 (PMC11004002; doi:10.1038/s41467-024-47411-4)
Supplement: Supplementary file 1 — Supplementary Information [file 41467_2024_47411_MOESM1_ESM.pdf]

Supplementary Materials for A 2D Chiral Microcavity based on Apparent Circular Dichroism

Tzu-Ling Chen,[1,2,6] Andrew Salij,[3,6] Katherine A. Parrish,[1,6] Julia K. Rasch,[1] Francesco Zinna,[4] Paige J. Brown,[3] Gennaro Pescitelli,[4] Francesco Urraci,[4] Laura A. Aronica,[4] Abitha Dhavamani,[5] Michael S. Arnold,[5] Michael R. Wasielewski,[3] Lorenzo di Bari,[4] Roel Tempelaar,[3*] Randall H. Goldsmith[1*]

[1]Department of Chemistry, University of Wisconsin-Madison, 1101 University Ave, Madison, WI 53706, USA, [2]Department of Photonics, National Yang Ming Chiao Tung University, 1001 Ta-Hsueh Road, Hsinchu, Taiwan, [3]Department of Chemistry, Northwestern University, 2145 Sheridan Rd, Evanston, IL 60208, USA, [4] Dipartimento di Chimica e Chimica Industriale, Università di Pisa, Via Giuseppe Moruzzi, 13, 56124 Pisa PI, Italy, [5]Department of Materials Science and Engineering, University of Wisconsin-Madison, 1415 Engineering Drive, Madison, WI 53706, USA, [6]These authors contributed equally to this work.

Section S1 CD degradation during microcavity fabrication
Section S2 HR Substrate Characterization
Section S3 Mueller Matrix Characterization of PTPO Films
Section S4 Microcavity Parameters and Finesse values
Section S5 Discussion of Cavity Enhancement
Section S6 Analysis of CD response of mechanically strained microcavity
Section S7 Theoretical treatment of microcavity chiral response
Section S8 Spatially- and temporally-resolved fluorescence measurements
Section S9 Theoretical Ising model for spatially varying CD signal
Section S10 Comparison with planar chirality in microcavity as demonstrated by Gautier et al
Section S11 Comparison of home-built and laboratory CD instrument
Section S12 Cavity Field Visualization
Section S13 Definitions of Circular Dichroism

Section S1 CD degradation during microcavity fabrication

To test whether the microcavity fabrication process changes the chiroptical signal of the PTPO film, we performed the same lamination process with PVA, but instead of using two HR substrates, we used one HR substate and one transparent coverslip. We found that the CD spectra were similar, but CD values were degraded by 10-15% relative to the same film measured on a single HR substrate. Thus, measured enhancement factors may be slightly underestimated.

Section S2 HR Substrate Characterization

The reflectivity of the HR substrates is shown in Figure S1.

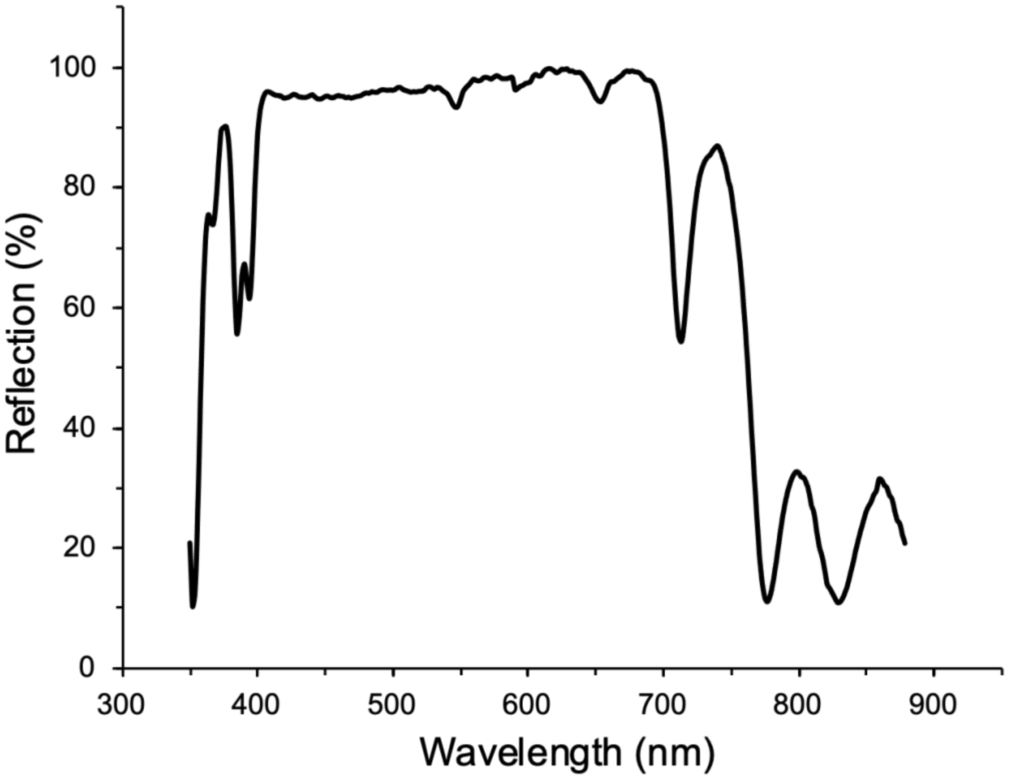

Figure S1. The reflection spectrum of the HR dielectric mirror was measured using a J. A. Woollam V-VASE ellipsometer. The measurements were performed with an incident angle of 20 degrees. To achieve a small measurement area, focusing probes were utilized on both the source and detector sides, enabling a spot size of 0.5 mm.

Section S3 Mueller Matrix Characterization of PTPO Films

Mueller matrix ellipsometry was performed on 300nm PTPO films deposited on an HR substrate (similar to Figure 1e). Mueller matrix elements were acquired on a JA Woollam RS2 ellipsometer equipped with focusing optics yielding a 300μm spot. Measurements were acquired at 20º from normal incidence at 450nm. A representative Mueller matrix from a spot near the center of the sample is shown below.

$$\begin{bmatrix} 1 & -0.337 & 0.133 & -0.044 \\ -0.351 & 0.519 & -0.003 & -0.104 \\ -0.137 & -0.017 & -0.374 & 0.125 \\ -0.053 & -0.102 & -0.114 & 0.100 \end{bmatrix}$$

In contrast, Mueller matrices acquired on HR substrates with no PTPO films showed near unity values along the diagonal and near zero values among the upper right and lower left 2x2 blocks, as expected.

Mueller matrix ellipsometry was performed on an 11x11 grid covering a 10x10 mm square area. As the M_{03} and M_{30} elements are most relevant to the ACD and asymmetric transmission properties of the material, we plot maps of M_{03}, M_{30}, $\frac{1}{2}(M_{03} + M_{30})$, and $\frac{1}{2}(M_{03} - M_{30})$ in Figure S2.

Mueller matrix ellipsometry was performed by Nina Hong at J.A. Woollam Company. We note that the Mueller matrix ellipsometry was performed in reflection-mode, for which the sign convention of the relation between M_ij and M_ji flips. Correspondingly, the terms $(M_{03} - M_{30})$ refers to the CD that is preserved upon sample flipping, while the terms $(M_{03} + M_{30})$ refers to the ACD which changes sign.

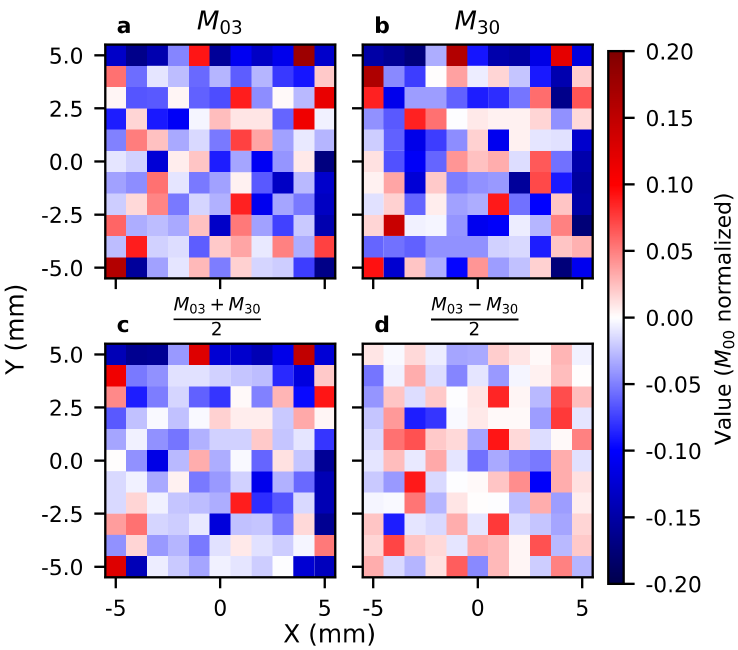

Figure S2. Spatial map of Mueller matrix elements $\boldsymbol{M_{03}}$ (a) and $\boldsymbol{M_{33}}$ (b) and combinations of elements $\frac{1}{2}(\boldsymbol{M_{03}} + \boldsymbol{M_{30}})$ (c) and $\frac{1}{2}(\boldsymbol{M_{03}} - \boldsymbol{M_{30}})$ (d) relevant for asymmetric response to circularly polarized light and ACD.

Section S4 Microcavity Parameters and Finesse values

For the empty cavities, where the spacer is only formed by the transparent (i.e. negligible absorbance in the visible regime) material polyvinyl acetate (PVA), the cavity finesse varies from 40 to 90. For microcavities fabricated with PTPO thicknesses of 100 nm and 50 nm, the cavity finesse is degraded due to the higher loss from the PTPO absorption. Consequently, the cavity finesse of 100 nm PTPO is lower than 50 nm.

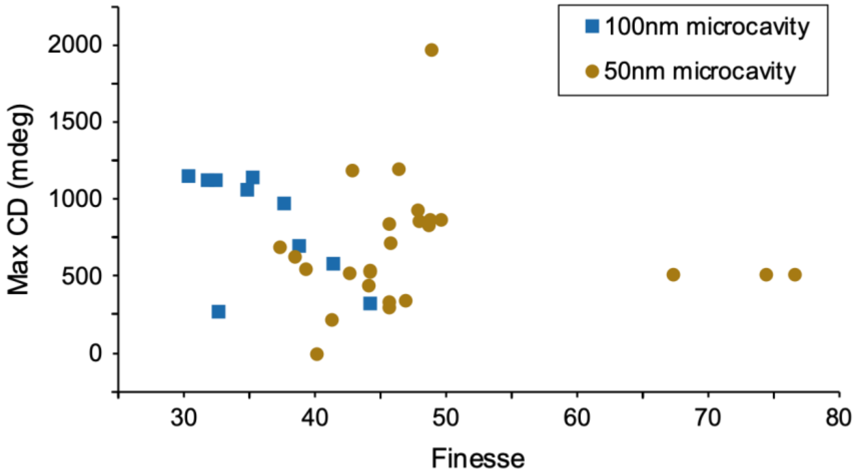

Figure S3. Distribution of maximum CD values and Finesse values for 100 nm and 50 nm microcavities.

Section S5 Discussion of Cavity Enhancement

In the main manuscript, we performed a qualitative estimate of the cavity enhancement of the chiroptical properties of the PTPO thin film. However, this estimate is difficult because of sample-to-sample and spot-to-spot variations in the intrinsic chiroptic response of the film, preventing a direct comparison between cavity-enhanced and non-enhanced samples. This section describes the use of an internal standard. Specifically, we rely on a small peak outside the high reflection regime of the DBR coating, known as the off-resonance peak or side-mode resonance. This peak is produced by the interference of light waves reflected between the surfaces of the DBR coating and the substrate or air layer, resulting in constructive and destructive interference patterns. Compared to the on-resonance peak, which occurs at the peak reflection wavelength of the DBR, the off-resonance peak experiences less amplification due to its origin in an imperfect constructive interference fringe outside the HR range. The off-resonance peak thus serves as an internal reference for calibration, allowing for a more precise quantification of the cavity enhancement.

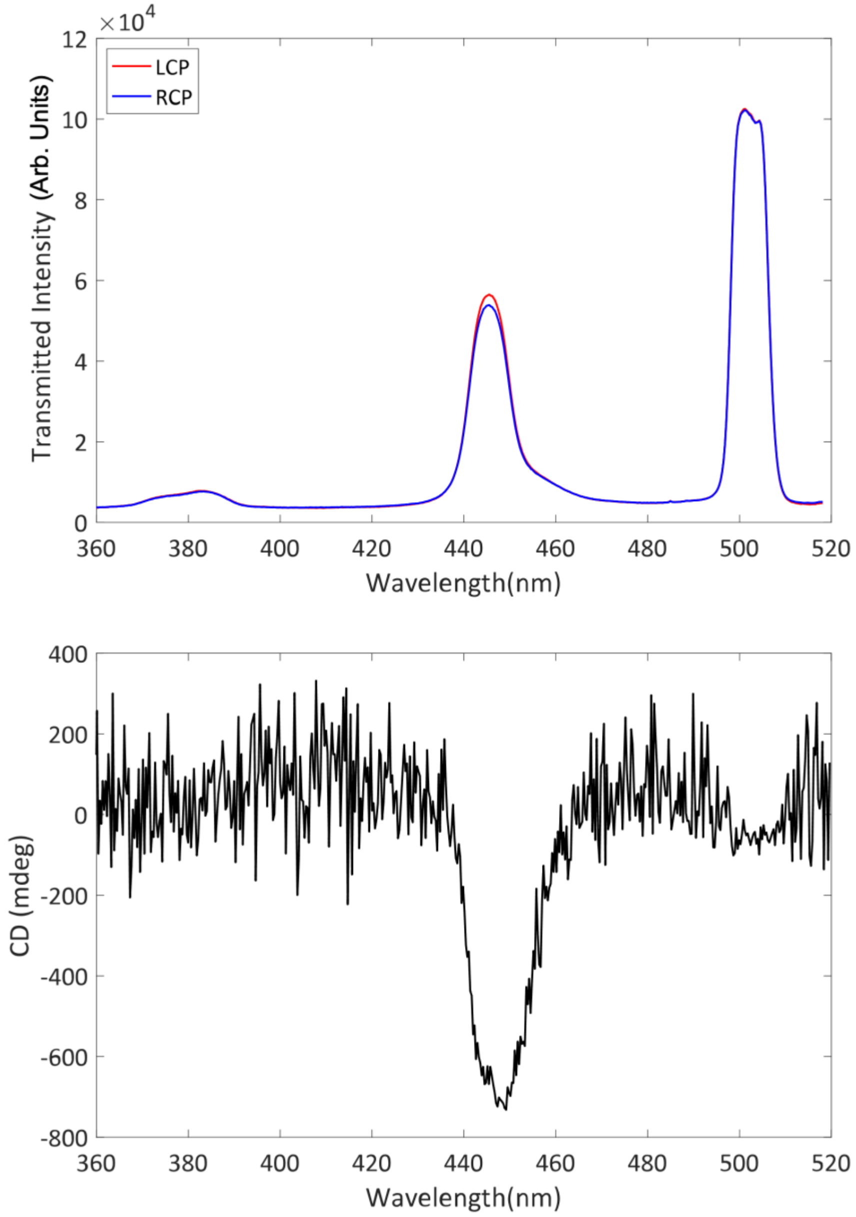

Figure S4. Larger spectral window of microcavity resonance shown in Figure 2c(i) showing additional minor spectral features.

Assuming that the enhancement of the chiroptic response is linearly proportional to the finesse, the ratio between the CD signal at the main on-resonance peak and the off-resonance peak takes the form,

$$\text{Ratio}_{\text{CD}} = \frac{F_{\text{on-resonance}}}{F_{\text{off-resonance}}} \frac{\text{ACD}_{\text{on}}}{\text{ACD}_{\text{off}}} = \frac{F_{\text{on-resonance}}}{\frac{\pi}{-\ln(R * T_{TF})}} \frac{\text{ACD}_{\text{on}}}{\text{ACD}_{\text{off}}} \sim \frac{\frac{\lambda}{\Delta\lambda_{\text{FWHM}}}}{\frac{\pi}{-\ln(0.6 * T_{TF})}} \frac{1600 \text{ mdeg}}{1300 \text{ mdeg}}$$

where $\text{ACD}_{\text{on(off)}}$ denotes the intrinsic chiroptic response on(off) resonance at a particular point of a particular sample, R is the mirror reflectivity, and T_{TF} is the transmittance of the thin film. Importantly, by comparing two spectral locations in the same measurement, the spot-to-spot variation is controlled for. The ratio $\frac{ACD_{on}}{ACD_{off}}$ can be estimated by looking at the CD signals of the film, Figure 1e, and has a value of 1600mdeg/1300mdeg. Further, since the microcavity is at the fundamental resonance and has length $L = \frac{\lambda}{2\text{n}}$, the usual expression for cavity finesse, $F_{\text{on-resonance}} = \frac{\lambda^2}{2\text{nL}\Delta\lambda_{\text{FWHM}}}$, reduces to $F_{\text{on-resonance}} = \frac{\lambda}{\Delta\lambda_{\text{FWHM}}}$, which is also the expression for the quality factor (Q) and can be extracted from each resonance. $F_{\text{off-resonance}}$ is determined based on the total loss in the cavity, and takes the form $\text{F}_{\text{off-resonance}} = \frac{\pi}{-\ln(R * T_{\text{TF}})}$, where R and T_{TF}take into consideration the losses from the mirror reflection and materials absorption, respectfully, for one pass through the system. The reflectivity at the off-resonance peak is $R = 0.6$.

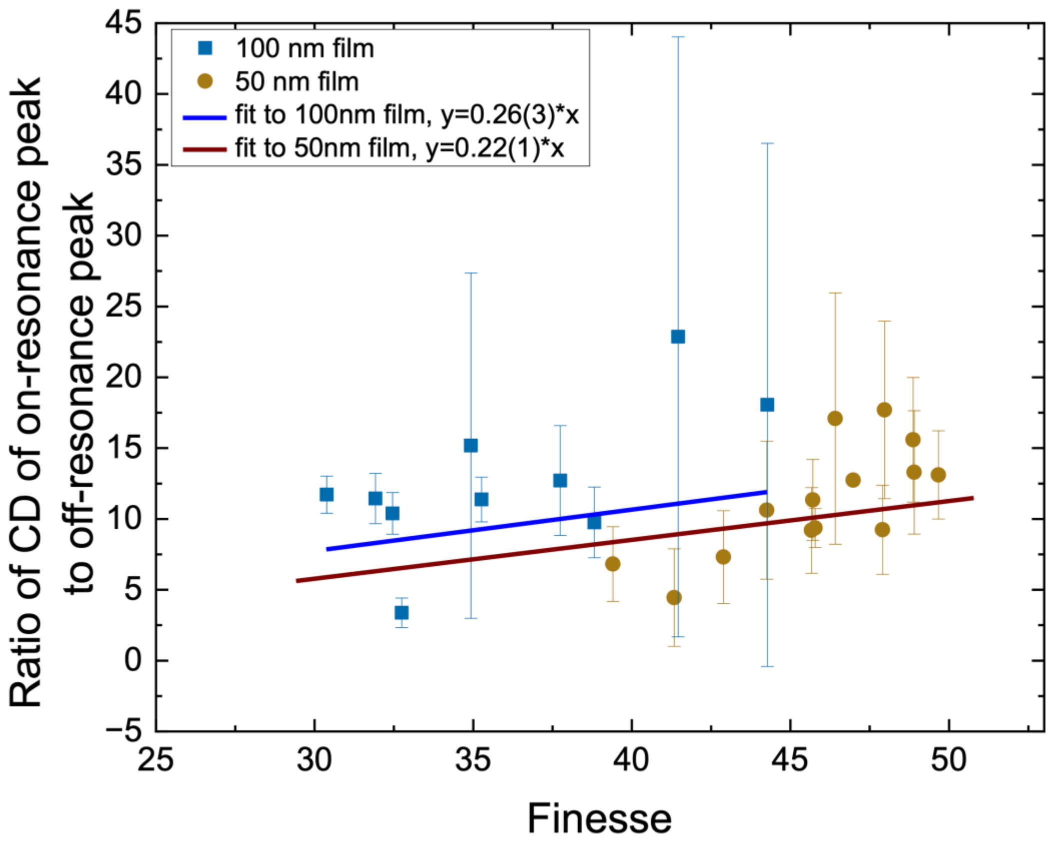

Figure S5. Plot of the ratio of CD at the on-resonance peak to the CD at the off-resonance peak vs the empirically measured cavity Finesse (F) of the on-resonance peak for 100 nm and 50 nm films along with linear fits; error bars indicate uncertainties σ_f.

We can also estimate T_{TF} from the PTPO thin film absorption spectra [4]. Measuring the absorption from a 100 nm thin film would be ideal, but the absorption is weak enough to make a quantitative measurement difficult. The absorbance of a 300 nm film at 375nm, the spectral position of the off-resonance peak, is $A = 0.21$. Assuming Beer's Law linearity, a 100 nm film is expected to have $A = 0.07$, corresponding to $T_{\mathrm{TF}} = 0.85$, and a slope between the CD ratio and the measured Q factor can be calculated as 0.26. Assuming this same linearity for a 50 nm film results in a predicted slope of 0.23 .

Figure S5 shows the empirical correlation between the CD Ratio of the on- and off resonance peaks with the observed finesse of the on-resonance peak for 100 nm and 50 nm films. Though there is significant uncertainty in the measurement, largely due to the difficulty in quantifying the very small CD values at the off-resonance peak, slopes for the 100 nm and 50 nm films are predicted to be 0.26 and 0.22, in excellent (though still qualitative) agreement with the calculation above.

This calculation serves as an additional line of evidence, in addition to what is offered in the main text, that the enhancement of the CD signal is linearly proportional to the finesse.

Section S6 Analysis of CD response of mechanically strained microcavity

To understand the contribution to the overall chiroptical response of the microcavity, particularly at higher angles, the angle dependent LB_{res} was determined both in "empty" microcavities and thin films without PTPO, and with only the PVA spacer, for comparison. Experimentally, Figure S5a-c, we observed that the LB_{res} increases at larger incident angle (a,b), and already exists, albeit weakly, at high angles even in samples with only one HR (c). In contrast to the real CD effect, which arises based on light-matter interaction (i.e. differential absorption), the LB_{res} is more akin to a non-ideality in the experimental setup.

Consequently, we built a theoretical model to predict how the LB_{res} will affect the observed CD spectra. Observations of a difference in response between LCP and RCP light is not unusual as dielectrostriction, the alteration of dielectric behavior due to the deformation of a material, can produce asymmetric optical behavior [5]. Here, we refer the interested reader to the relevant literature on the difference and interplay between electrostriction, the deformation of a material under electric stress, and dielectrostrictive effects [5,6]. Particularly for our HR substrates, which are DBR coated on a very thin coverslip (~0.18 mm), stress or strain are applied during the deposition process, microcavity fabrication, or in the mirror mounting.

The theoretical model uses a double DBR stack of SiO_2/Ta_2O_5 modelled as initially isotropic dielectrics with refractive indices *n* of 1.5 and 2.2, respectively. For simplicity, the DBR is assumed to lack dispersion as the dielectric behavior is largely constant in the wavelength region of interest.

A shear stress σ_{xy} is then applied to the entire structure, producing shear strains modelled via Hooke's Law, with[7]

$$\begin{pmatrix} u_{xx} \\ u_{yy} \\ u_{zz} \\ 2u_{yz} \\ 2u_{zx} \\ 2u_{xy} \end{pmatrix} = \frac{1}{E}\begin{pmatrix} 1 & -\nu & -\nu & 0 & 0 & 0 \\ -\nu & 1 & -\nu & 0 & 0 & 0 \\ -\nu & -\nu & 1 & 0 & 0 & 0 \\ 0 & 0 & 0 & 2(1+\nu) & 0 & 0 \\ 0 & 0 & 0 & 0 & 2(1+\nu) & 0 \\ 0 & 0 & 0 & 0 & 0 & 2(1+\nu) \end{pmatrix}\begin{pmatrix} \sigma_{xx} \\ \sigma_{yy} \\ \sigma_{zz} \\ \sigma_{yz} \\ \sigma_{zx} \\ \sigma_{xy} \end{pmatrix}$$

where E is Young's modulus for an isotropic material, ν is Poisson's ratio, σ_{ij} are stress tensor elements, and u_{ij} are strain tensor elements. For the DBR material parameters, we use the literature values for thin films deposited on silicon substrates using the one-substrate method, giving E = 91 GPa, ν = 0.19 for SiO_2 and E = 138 GPa, ν = 0.33 for Ta_2O_5 [8]. From the resulting strain on SiO_2 and Ta_2O_5 , the dielectric tensor is then modified as [5,6]

$$\epsilon_{f,ij} = \epsilon_{0,ij} + a_1 u_{ij} + a_2 \mathrm{Tr}\{\mathbf{u}\}$$

$$a_1 = -\frac{2}{5}(\epsilon - 1)^2$$

$$a_2 = -\frac{1}{3}(\epsilon - 1)(\epsilon + 2) + \frac{2}{15}(\epsilon - 1)^2 .$$

Here, the electrostriction parameters (a_1,a_2) are predicted using Shkel and Klingenberg's theory of the electrostriction isotropic solid and are correspondingly constant as the refractive index is treated as constant for the DBR materials [9]. To reproduce observed chiral signals, we parameterize high stresses of 100MPa, which should be understood not as precise predictions for the internal stress of the DB. Rather, the model and its parameters should be understood as describing ideal Hooke's law behavior for a perfectly constructed mirror. That actual stresses on the DBR are almost certainly lower than the parameterized value of 100MPa.

In Figure S6d-e, for a given resonance, the bisignate behavior is maintained for the cavity while a single mirror is monosignate, as is experimentally observed. For the bare mirror, Figure S5c,f, at sufficiently high angles there is a weak CD signal observed in both theoretical and experimental spectra. From simulations, we have observed that the circular discrimination occurs in regions of imperfect reflectance such as the edge of the spectral region where the DBR is effective or where there is slight interference between the target wavelengths of the DBR reflectors. For the latter phenomenon, the DBRs have multiple sets of periods spaced to target different wavelengths in order to have high reflectance over a bandwidth larger than could be supported using a single periodic array, but this property produces small bandwidth regions within the total spectral region of reflectance where interferences between multiple modes weaken total reflectance.

For the microcavity, Figure S5a,b,d,e, there is a shift in the resonant frequencies for LCP and RCP polarizations, resulting in bisignate behavior. These experimental effects are well-reproduced by our theoretical calculations and are due to strain-induced changes to the reflectance and not actual absorption of light in contrast to ACD. The CD also does not change sign upon shifting the direction of propagation. In the main text, we assert that this behavior is a kind of extrinsic 2D chirality that treats opposite signs of incidences identically but which would change sign upon sample flipping, which raises the question as to why opposite directions of propagation could

produce identical signals. Such an understanding would prove accurate for a single mirror, but for both mirrors that are facing one another, the net system does not change upon sample flipping, answering this apparent discrepancy. At low angles, these effects are much weaker than the chiral response of an embedded ACD-active system, but they can come to dominate at sufficiently large angles. This issue, as well as the key advantages of using microcavities at normal incidence, motivated focusing this effort on normal incidence.

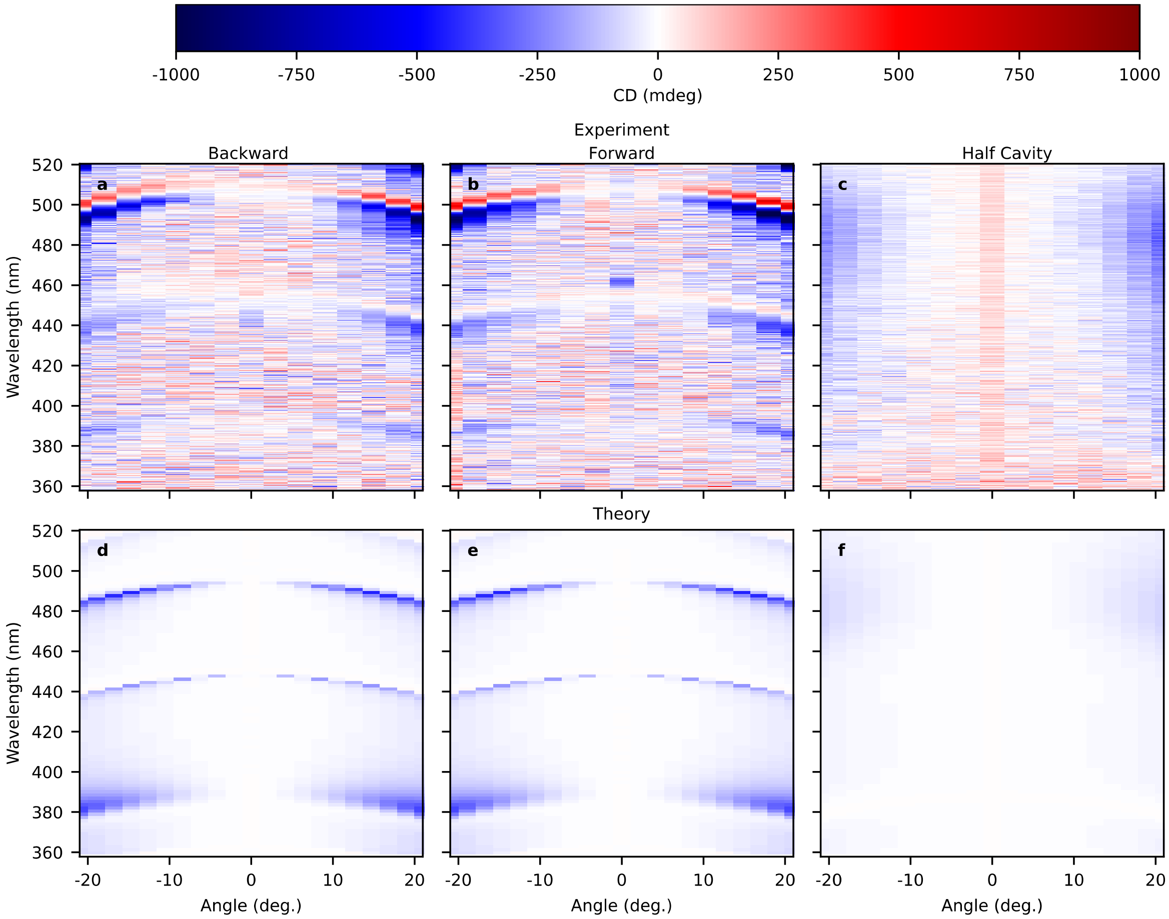

Figure S6. Experimental and theoretical characterization of hybrid DBR mirrors under shear stress. a-c) Experimental bare cavity with forwards (a) and backwards (b) propagation and open cavity mirror (c) CD. d-f) Theoretical bare cavity with forwards (d) and backwards (e) propagation and open cavity mirror (f) CD. Mirrors modelled as DBR stacks of periodicity 4 with target wavelengths of maximum reflectance at 385nm and 470 nm. Ideal mirrors consist of alternating isotropic nondispersive dielectrics for SiO_2 (n = 1.5, E = 91 GPa) and Ta_2O_5 (n = 2.2, E = 138 GPa)[7]under 100MPa of shear (xy) stress. The shear stress is applied to the mirror in orientation, from top to bottom, of SiO_2/Ta_2O_5 (385 nm, 4 periods), SiO_2/Ta_2O_5 (470nm, 4 periods) such that the entire system is symmetric to flipping the cavity as both mirrors are oriented inwards.

Section S7 Theoretical treatment of microcavity chiral response

To obtain parameterization for the transition dipoles of PTPO, a combined approach of time-dependent density functional theory (TD-DFT) and a Lorentz oscillator model was implemented.

That is, we first determined initializations for the dipole operators via TD-DFT, but due to uncertainty and challenges in accurately modelling thiophenes, we refined such results against experimental data in a series of successively more restrictive optimizations to solution absorption spectra, thin film absorption spectra, and thin film CD spectra, following the approach from Ref.[10]

Section S7.1 TD-DFT

Using QChem 5.3 [11], we performed geometry optimization of single molecules of PTPO *sans* sidechains *in vacuo* with a functional/basis combination of ωB97X-D/6-311G(d,p) [12,13], where the exchange-correlation function was chosen for its effectiveness in modelling thiophenes [14]. Subsequently, we performed time-dependent density functional theory (TD-DFT) on the resulting optimized geometries using CAM-B3LYP/def2-TZVPPD [15,16].

Multiple thiophene ring conformations were calculated, and the stable configuration, being where the carbonyl by the thiophene ring is *trans* to the sulfur, was ultimately chosen for further analysis. We focus our analysis on the results from optimization in vacuum, which were broadly similar to those that were solvated in chloroform via a polarizable continuum model with the integral equation formalism (IEF-PCM).[17]

Section S7.2 Solution spectra

To obtain parameters for the transition dipoles, we began with the parameterization obtained via TD-DFT and then sequentially refined with respect to experiment. Specifically, the seven largest transition dipoles in the spectral region of interest were the initializations for an optimization between experimental spectra [4] and a Lorentz oscillator model for absorption [10]. Here, absorption has the form

$$A \propto \omega \sum_n \omega_n \mu_n^2 \frac{\gamma_n \omega}{(\omega_n^2 - \omega^2)^2 + \gamma_n^2 \omega^2}$$

with μ_n being a dipole strength, ω_n being the transition frequency for the *n*th excited state, and $\gamma_n = \gamma\omega_n$ being a damping factor. To account for vibrational modes, we fit constants for damping γ, the Huang-Rhys factor λ, and vibrational energy splitting ΔE_{vib} within constraints chosen for organic molecules. With refined parameters from normalized absorption spectra in solution, we found consistency between the model and experiment, Figure S6. In doing so, we obtained excitation energies and dipole strengths, Table S1, for the seven excited states that are redshifted

from the TD-DFT results. That noted, the general dipole strengths and energy spacings between the states proved broadly consistent with computational parameters.

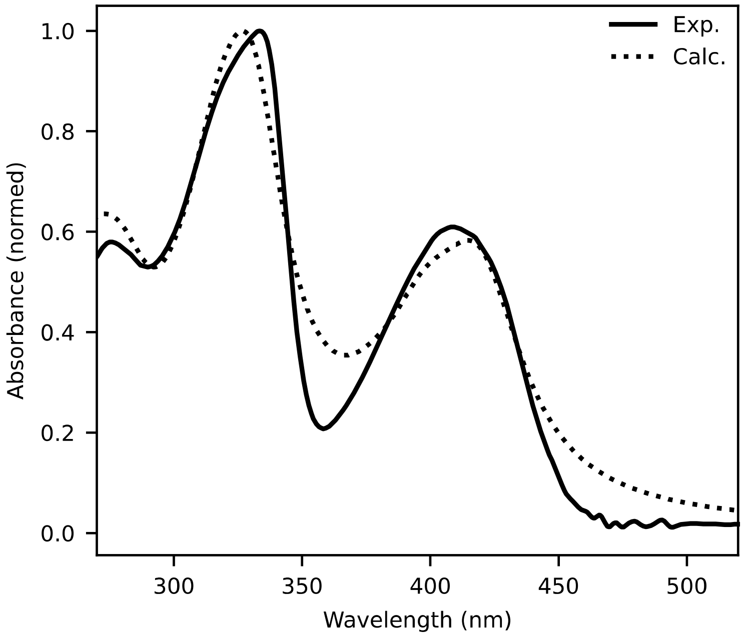

Figure S7. Comparison between normalized absorbances for previous experimental[4] and theoretical spectra. Experimental spectra from (*S,S*)-PTPO solvated in chloroform. Theoretical spectra fit to a Lorentz oscillator model with constants γ = 0.098, ΔE_{vib} = 0.161eV, and λ = 0.7629.

E_n(eV) TDDFT	E_n (eV) sol. fit	E_n(eV) tf fit	μ_n (D) TDDFT	μ_n(D) fit	θ_n (deg.) TDDFT	θ_n (deg.) fit
3.54	2.96	2.69	8.70	6.21	0	-10.60
4.31	3.75	3.77	5.24	7.86	6.53	7.32
4.90	4.42	4.37	3.86	4.56	102.74	111.35
4.97	4.57	4.57	3.38	2.72	43.61	30.08
5.34	4.68	4.68	2.81	2.85	38.63	33.47
5.91	5.48	5.48	3.57	4.68	92.67	79.96
6.07	5.61	5.61	2.89	3.33	10.44	-.42

Table S1. Parameters from TDDFT calculations (vacuum) and from fitting. Other relevant parameters to lineshapes include γ = 0.098, ΔE_{vib} = 0.161eV, and λ = 0.763, and crystal unit cell volume v = 1.93 nm^3.

Section S7.3 Thin Film and Cavity Spectra

Within a film, interface effects and internal coherences can become relevant. In order to properly and consistently account for these effects, both thin film and cavity spectra were treated with the Scattering Matrix Method (SMM) using a modified version of open-source package PyLlama[18] in

conjunction with our own post/pre-processing in Python 3.10 (numpy (1.22.3) [2], scipy (1.7.3) [3]. In the SMM [19], Maxwell's equations are solved for propagation in one dimension, ensuring that any coherent effects due to internal reflection are fully simulated.

At this level of theory, only the dielectric tensor varies between media with the permeability tensor treated as unity and optical activity (i.e, magneto-electric coupling) tensors are null. Both experimentally and theoretically, we obtain an enhancement of the total CD response, indicating that the inversion anti-symmetry of PTPO preserves chiroptical information within cavity cycles.

Here, we will follow the notation and give an overview of the algorithm used in PyLlama and reported in the corresponding manuscript[18]. Specifically, we consider a one-dimensional system of optical elements from a half-infinite space of air, an ordered set of dielectric objects in the system, and then another half infinite space of air. To implement, a scattering matrix S is calculated for each optical element that relates incoming, reflected, transmitted, and back-scattered electromagnetic waves of *s* and *p* polarization such that

$$\begin{pmatrix} E_{p,\rightarrow}(z_f) \\ E_{s,\rightarrow}(z_f) \\ E_{p,\leftarrow}(z_0) \\ E_{s,\leftarrow}(z_0) \end{pmatrix} = \mathbf{S} \begin{pmatrix} E_{p,\rightarrow}(z_0) \\ E_{s,\rightarrow}(z_0) \\ E_{p,\leftarrow}(z_f) \\ E_{s,\leftarrow}(z_f) \end{pmatrix}$$

with arrows indicating wave direction (right/left indicating forwards/backwards in *z*) and for initial incidence coordinate z_0 and final coordinate z_f for propagation in the *xz* plane. In such a scheme, *p* (parallel to plane of incidence) polarization corresponds to *x* and *s* (perpendicular) to *y* for the plane-polarized incident radiation. Overall propagation from source to transmission measurement and arrangement of optical elements here are in the positive *z* direction, and we allow the incident light wave to take some incident angle with respect to normal from the film in the *xz* plane. To calculate **S**, one begins with an ordinary differential equation that recasts Maxwell's equations for a wave propagating in the *z* direction through uniform layers [20] given as

$$\frac{\partial \vec{\boldsymbol{\psi}}(z)}{\partial z} = \frac{i\omega}{c}\boldsymbol{\Delta}(z)\vec{\boldsymbol{\psi}}(z)$$

here termed Berreman's equation, and where

$$\vec{\boldsymbol{\psi}} = (E_x \quad H_y \quad E_y \quad -H_x)^T$$

$$\boldsymbol{\Delta}(z) = \begin{pmatrix} -\frac{k_x}{k_0}\frac{\epsilon_{zx}}{\epsilon_{zz}} & 1-\frac{k_x^2}{k_0^2\epsilon_{zz}} & -\frac{k_x}{k_0}\frac{\epsilon_{zy}}{\epsilon_{zz}} & \frac{k_x}{k_0\epsilon_{zz}} \\ \epsilon_{xx}-\frac{\epsilon_{xz}\epsilon_{zx}}{\epsilon_{zz}} & -\frac{k_x}{k_0}\frac{\epsilon_{xz}}{\epsilon_{zz}} & \epsilon_{xy}-\frac{\epsilon_{xz}\epsilon_{zy}}{\epsilon_{zz}} & \frac{\epsilon_{xz}}{\epsilon_{zz}} \\ 0 & 0 & 0 & 1 \\ \epsilon_{yx}-\frac{\epsilon_{yz}\epsilon_{zx}}{\epsilon_{zz}} & -\frac{k_x}{k_0}\frac{\epsilon_{yz}}{\epsilon_{zz}} & \epsilon_{yy}-\frac{k_x^2}{k_0^2}-\frac{\epsilon_{yz}\epsilon_{zy}}{\epsilon_{zz}} & \frac{\epsilon_{yz}}{\epsilon_{zz}} \end{pmatrix}$$

with k_i being the wavevector in direction i with total magnitude k_0 and ε_{ij} being elements of the dielectric tensor for a material layer. The solution to the initial differential equation for propagation between two interfaces at coordinates z_i and z_{i+1} through layer i is

$$\vec{\boldsymbol{\psi}}(z_{i+1}) = \exp(ik_0 l_i \boldsymbol{\Delta}_i)\,\vec{\boldsymbol{\psi}}(z_i) = \mathbf{P}_i \mathbf{Q}_\mathbf{i} \mathbf{P}_i^{-1}$$

where l_i is the layer thickness, P_i is a matrix of eigenvectors of Δ_i, and

$$\mathbf{Q}_i = \exp(ik_0 l_i \mathbf{Q}_{\Delta,i})$$

where $\mathbf{Q}_{\Delta,i}$ is a diagonal matrix of the eigenvalues of Δ_i. To account for various incidence angles, we note that

$$\begin{pmatrix} E_x \\ H_y \\ E_y \\ -H_x \end{pmatrix} = \begin{pmatrix} \cos\theta_e & 0 & \cos\theta_e & 0 \\ n_e & 0 & -n_e & 0 \\ 0 & 1 & 0 & 1 \\ 0 & n_e\cos\theta_e & 0 & -n_e\cos\theta_e \end{pmatrix} \begin{pmatrix} E_{p,\rightarrow} \\ E_{s,\rightarrow} \\ E_{p,\leftarrow} \\ E_{s,\leftarrow} \end{pmatrix}$$

for the direct transmission from(into) entry(exit) media with refractive indices $\boldsymbol{n_e}$ and angles of incidence $\boldsymbol{\theta_e}$ [21]. Here, both entry and exits are treated as air for both half-infinite spaces. After ordering the partial waves such that the two solutions travelling forwards in the z direction generally precede the two travelling in the $-z$ direction, the relationship between the electric fields between two subsequent layers is

$$\mathbf{P}_{i+1} \begin{pmatrix} E_{i+1,\rightarrow}(z_{i+1}) \\ E_{i+1,\leftarrow}(z_{i+1}) \end{pmatrix} = \mathbf{P}_i \mathbf{Q}_i \begin{pmatrix} E_{i,\rightarrow}(z_i) \\ E_{i,\leftarrow}(z_i) \end{pmatrix}$$

For simplicity of notation, here and for much of the remainder of this section we switch to a block form of the above matrices and vectors such that each electric field element accounts for the two solutions to Berreman's equation, turning the 4-by-4 matrices into 2-by-2 block matrices. Upon algebraic rearrangement, a layer scattering matrix $\mathbf{S}_{i,i+1}$
can be constructed to connect the incoming and outgoing waves such that

$$\begin{pmatrix} E_{i+1,\rightarrow}(z_{i+1}) \\ E_{i,\leftarrow}(z_i) \end{pmatrix} = \mathbf{S}_{i,i+1} \begin{pmatrix} E_{i,\rightarrow}(z_i) \\ E_{i+1,\leftarrow}(z_{i+1}) \end{pmatrix}$$

To combine the scattering matrices of individual layers, standard matrix multiplication between the matrices does not properly account for the coupling between incoming and outgoing waves. Rather, one applies the star product [22,23], defined here [Note [1]] as

$$\mathbf{S}^{(A)} \star \mathbf{S}^{(B)} = \begin{pmatrix} \mathbf{S}_{00}^{(B)}\mathbf{D}\mathbf{S}_{00}^{(A)} & \mathbf{S}_{01}^{(B)} + \mathbf{S}_{00}^{(B)}\mathbf{D}\mathbf{S}_{01}^{(A)}\mathbf{S}_{11}^{(B)} \\ \mathbf{S}_{01}^{(B)} + \mathbf{S}_{11}^{(A)}\mathbf{S}_{10}^{(\mathrm{B})}\mathbf{D}\mathbf{S}_{00}^{(A)} & \mathbf{S}_{11}^{(A)}\left(\mathbf{I} + \mathbf{S}_{10}^{(B)}\mathbf{D}\mathbf{S}_{01}^{(A)}\right)\mathbf{S}_{11}^{(B)} \end{pmatrix}$$

[1] We order the waves of the output electric field vector differently here than in the common convention, so the product to connect adjacent scattering vectors is different than the standard form of the Redheffer star product. Here, we follow the convention used by PyLlama [18] which we used for SMM calculations.

$$\mathbf{S}^{(\alpha)} = \begin{pmatrix} \mathbf{S}_{00}^{(\alpha)} & \mathbf{S}_{01}^{(\alpha)} \\ \mathbf{S}_{10}^{(\alpha)} & \mathbf{S}_{11}^{(\alpha)} \end{pmatrix}; \qquad \mathbf{D} = \left(\mathbf{I} - \mathbf{S}_{01}^{(A)}\mathbf{S}_{10}^{(B)}\right)^{-1}.$$

As the star product is associative, successive applications of it connect all the scattering matrices into a single matrix as given as the start of this section. From **S**, one obtains the reflection and transmission coefficients for the entire system

$$\mathbf{R}_{ps} = \begin{pmatrix} r_{pp} & r_{ps} \\ r_{sp} & r_{ss} \end{pmatrix} = \begin{pmatrix} S_{20} & S_{21} \\ S_{30} & S_{31} \end{pmatrix}$$

$$\mathbf{T}_{ps} = \begin{pmatrix} t_{pp} & t_{ps} \\ t_{sp} & t_{ss} \end{pmatrix} = \begin{pmatrix} S_{00} & S_{01} \\ S_{10} & S_{11} \end{pmatrix}$$

To determine spectra for an arbitrary polarization, the transmission matrix **T** presents an effective tool. Letting xz be the plane of incidence, transmitted intensity of each polarization is

$$|\mathbf{T}\,\vec{\mathbf{j}}|^2 = |\vec{J}_T|^2 = \begin{pmatrix} t_a^2 \\ t_b^2 \end{pmatrix} = \begin{pmatrix} I_a \\ I_b \end{pmatrix}$$

where $\vec{\mathbf{j}}$ is some incident Jones polarization vector and with the measured intensity being the sum of I_a and I_b for orthogonal polarizations a and b. For left- (l) and right-handed (r) polarization, one transforms the basis as

$$\begin{pmatrix} t_{ll} & t_{lr} \\ t_{rl} & t_{rr} \end{pmatrix} = \begin{pmatrix} 1 & 1 \\ -i & i \end{pmatrix}^{-1} \begin{pmatrix} t_{pp} & t_{ps} \\ t_{sp} & t_{ss} \end{pmatrix} \begin{pmatrix} 1 & 1 \\ -i & i \end{pmatrix}$$

in the convention that the Jones vector of right-handed polarization is $\frac{1}{\sqrt{2}}\begin{pmatrix} 1 \\ i \end{pmatrix}$.

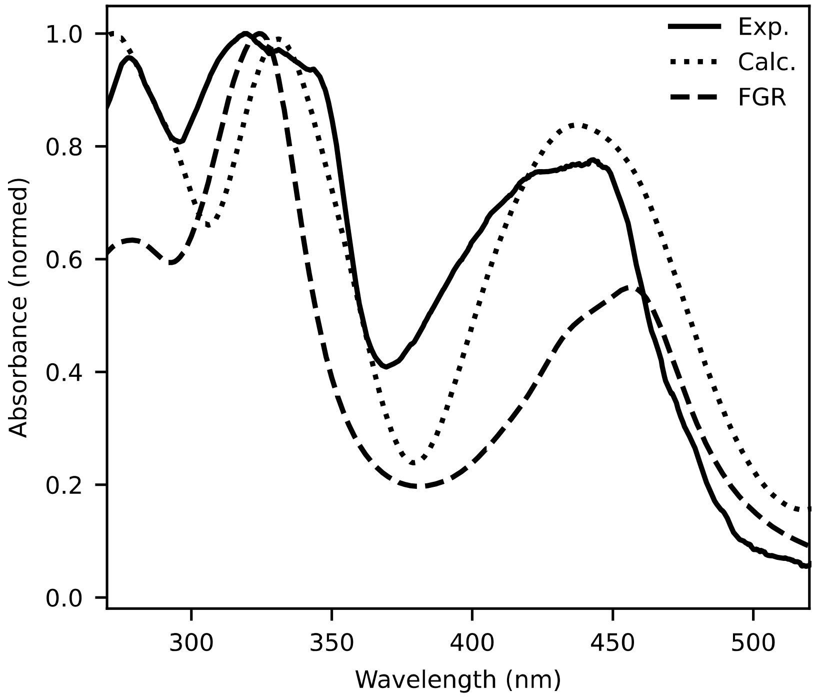

Figure S8. Comparison between normalized absorption spectra for PTPO thin films from previous experiment[4] and theoretical fit. Fit determined via optimization of results from SMM.

To implement the SMM, we modified the open-source code for PyLlama [18] to allow for spectral dispersion of dielectric tensors. We model the dielectric tensor via transition dipole oscillators at $k = 0$, that is, without spatial dispersion. In our model, all angle-dependent chiroptical response calculated results from wavevector-dependent effects propagating eigenmodes and the continuity of Maxwell's equations, *not* an intrinsic spatial dispersion of the system. While PTPO does possess an inversion center due to its aliphatic chains, we posit that these chains predominantly affect the orientation and stacking of the thiophene rings and not the transitions themselves as both linear absorption and CD spectra of the thin films are reproduced via computational methods omitting said chains. Omitting the side chains, the visibly-absorptive fragment of PTPO possesses an inversion center, so electric dipole forbidden transitions would constitute the majority of angular dispersion of the dielectric tensor with k^2 dependence [24]. Evidently, such effects, presumably weak in any case, prove unnecessary to understanding the chiroptical response, and the more basic

considerations of the incident light ray's pathlength and angle formed relative to the transition dipoles predominate.

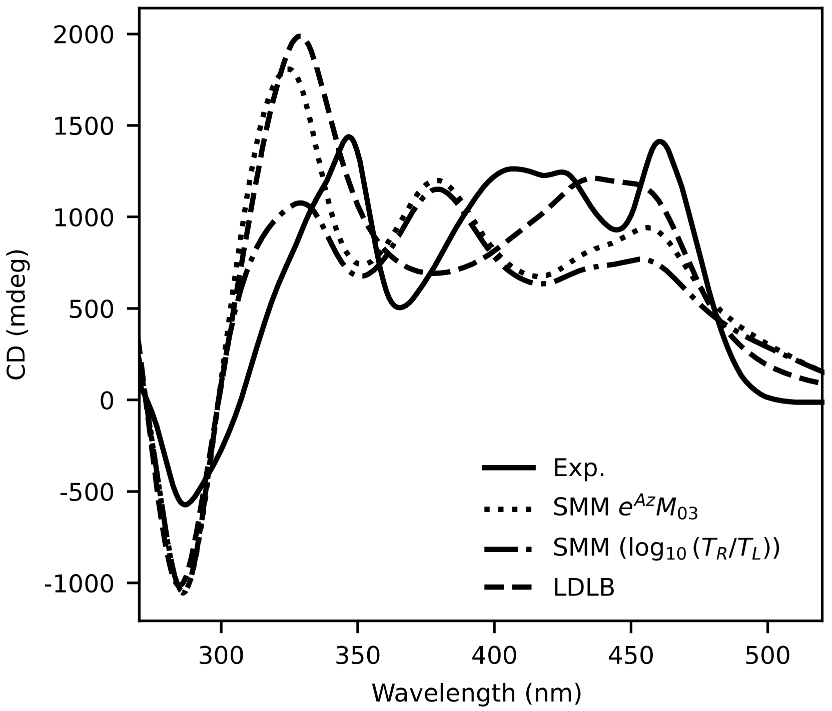

Figure S9. Comparison between CD PTPO thin films from previous experiment[4] and theoretical fit. LDLB is an analytic treatment of Mueller calculus to second order that omits internal reflection and interface effects. Fit parameters identical to those in Figure S7. Two SMM plots demonstrate the different definitions of CD in terms of the normed one analogous to LDLB and the un-normed, directly observed signal.

For the fitting of parameters to the PTPO thin films, we considered a single layer of 300 nm-thick PTPO oriented in the *xy* plane. Here, we used measurements over a broader spectral range than the scans for the cavities in the main text so as to be able to characterize the low wavelength peaks whose parameterization matters for the ACD response in the 400-500 nm region. A thicker 300 nm film as opposed to a 100 nm films was used for the parameterization due to its greater signal to noise for absorption and CD, thus providing a better data set for parameter optimization. To define an absorbance of light polarizations, we averaged the negative logarithms of the transmissions of *l* and *r*-polarized vectors.

For the fitting of parameters, the fits for solution were taken as initializations, and a modest solvent-crystal energy shift was allowed for the lowest three excited states, and we obtained close agreement between calculated spectra and experimental results [4], Figure S7.

All transition dipole parameters except for direction were not allowed to vary after fitting to solution spectra, but the dipoles were projected into the *xy*-plane and allowed to vary in their angle

with the x-axis. As ACD occurs due to the angles *between* transition dipole moments, said dipoles were allowed to be perturbed from their initial directions in order to determine CD, defined as

$$\mathrm{CD} \approx e^{Az}\mathbf{M}_{03}$$

where M is the Mueller matrix from transmission through the sample and

$$-Az = \frac{1}{4}\mathrm{Tr}\{\mathrm{logm}(\mathbf{M})\}$$

The Mueller matrix encodes the same information as the transmission matrix determined from SMM while separating out all net polarization effects. It is calculated in the xy basis as [25]

$$\mathbf{M} = \mathbf{A}\left(\mathbf{T}_{xy} \otimes \mathbf{T}_{xy}^{*}\right)\mathbf{A}^{-1}$$

$$\mathbf{A} = \frac{1}{\sqrt{2}}\begin{pmatrix} 1 & 0 & 0 & 1 \\ 1 & 0 & 0 & -1 \\ 0 & 1 & 1 & 0 \\ 0 & i & -i & 0 \end{pmatrix}$$

The results from the SMM, Figure S8, are in good agreement with experimental data as well as with our previous perturbative method of approximating ACD [10] shown as LDLB. The rationale for the norming of CD with regards to absorption is to produce a direct comparison of CD to LDLB, for which mean absorption has no effect. For experimental measurements and theoretical comparisons besides Figure S8, the more natural definition that is directly in terms of a ratio of logarithms of transmissions is used.

In the main text, we discuss angular chiroptical within the cavity extensively, but such behavior extends to the thin film outside of the cavity. SMM calculations of both 100 and 300 nm PTPO thin films using previous parameterizations are shown below, Figure S9. Noting particular attention to the spectral region near 460 nm, SMM techniques reproduce a bare film CD magnitude of 770 mdeg at normal incidence for the 300 nm thin film (Figure S9b), a roughly fourfold increase from the corresponding region for the 100 nm film (CD = -210 mdeg). This magnitude increase corresponds to a slightly greater than linear increase of CD with pathlength with an additional contribution to CD from interference due to the film thickness times the isotropic refractive index being on the order of the incident radiation, which is also the origin of the highly chiroptical behavior around 380 nm in Fig S9b not mimicked in Figure S9a.

Comparison between the TD-DFT parameters and the fits obtained from both solution and thin film spectra yields general agreement between all excitations, Table S1. The core distinction is

between the energies, though with the limitations of DFT it is not particularly surprising that the energies are overestimated by TD-DFT compared to experimental spectra.

As noted in the main text, the FP cavity itself produces a significant contribution to the measured chiral signal. As discussed in greater detail above, shear strain on the mirrors results in a difference between the reflectance for each circular polarization, contributing to a measured chiroptical transmission (Figure S5). In brief, the microcavities without PTPO exhibit a CD response at oblique incidence which has a sign that is invariant upon switching from forward to backwards propagation (forwards illumination is shown in Figure 3c and 3f, with results for both directions shown in Figure S5). Since the mirrors are facing inwards to one another, any anisotropy of the single mirror is literally mirrored by its twin on the other side of the cavity.

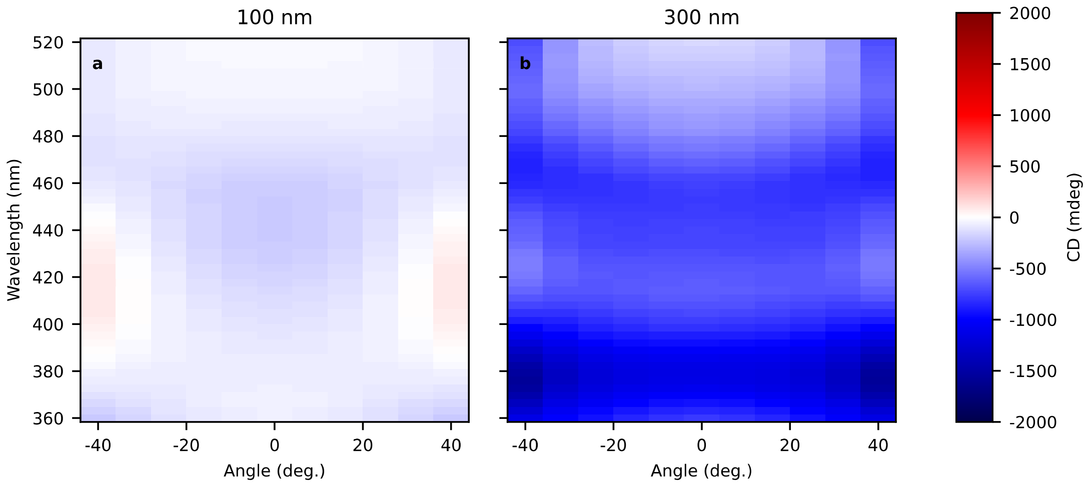

Figure S10. Theoretical SMM angle-resolved CD for PTPO thin films of 100 nm (a) and 300 nm (b) thicknesses. Parameterization identical to that in the theoretical fit in Figure S8 but for flipped orientation.

While the empty microcavities present some chiral artifacts at oblique incidence that are presumably due to strain induced in their fabrication (Figure 3c), we observe a strong cavity-enhancement of the chiral response of PTPO independent of strain effects at and around normal incidence (Figure 3a,b). The normal incidence response can essentially be thought of as the ACD-active thin film having its effective pathlength enhanced via the mirrors. As ACD increases proportionally with interaction length [26], the cavity enhances the chiral response proportionally to its finesse. Keeping angle constant, and varying wavelength there exists chiroptical sign inversions in Figure 3 that we understand as due entirely to Lorentz-reciprocal classical behavior. Specifically, there are two relevant processes: one within resonant modes of the cavity and the other within the PTPO films themselves. For the former, at high angles the LCP and RCP resonances are shifted from one another, resulting in a bisignate chiral response of the entire cavity system (top frequency region of Figure S5ab). For the native response of PTPO, as incident angle changes, so too does the angles that the propagating waves through PTPO make with the electronic transition dipoles. Furthermore, greater angles of incidence result in longer effective pathlengths through the PTPO film, and thus enhanced chirality even outside of the cavity (Figure S9).

Section S8 Spatially- and temporally-resolved fluorescence measurements

Picosecond time-resolved fluorescence data, Figure S10, were collected using a commercial direct-diode-pumped 100 kHz amplifier (Spirit 1040-HE, Spectra-Physics), producing a fundamental beam of 1040 nm (350 fs, 12 W) which was attenuated and used to pump a non-collinear optical parametric amplifier (Spirit-NOPA, SpectraPhysics) capable of delivering tunable, high-repetition-rate pulses with pulse durations as short as sub-20 fs. The pump beam was routed through a Nikon Eclipse Ti-U microscope and focused using a 40x microscope objective. The samples were excited with 400 nm, ~1 nJ laser pulses. Fluorescence was detected using a Hamamatsu C4780 Streakscope as previously described. All data were acquired in single-photon-counting mode using the Hamamatsu HPD-TA software. The temporal resolution, given by the instrument response function (IRF), was approximately 3% of the sweep window, with the shortest time resolution being ~30 ps.

The data were worked up by averaging over the emission bands, then the averaged spectra was fit with a biexponential decay. Both spots were fit simultaneously, with the lifetimes help constant between the two spots. The amplitude of each species was allowed to vary between spots. In the 10 ns window the shorter time constant was fixed to the IRF of window, 200 ps. The amplitude of each species varies from spot to spot, and in the larger window it appears though the faster species dominates for Spot 1 whereas for Spot 2 the longer-lived species is the dominant species.

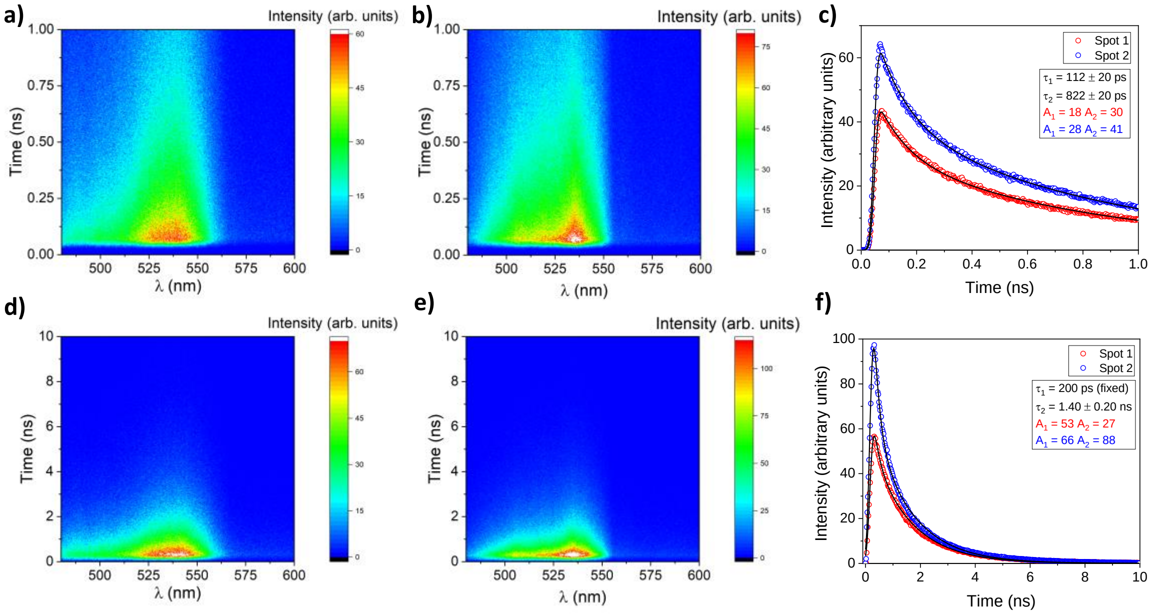

Figure S11**.** TRF spectra of the cavity at two different spots, Spot 1 (a & d) and Spot 2 (b & e). The lifetimes were measured over a 1 ns window (a & b) and a 10 ns window (d & e). (c & f) The signal for each spot was averaged from 500 nm to 550 nm and then both spots were fit simultaneously with a biexponential decay. The time constants were kept constant between the two spots but the amplitude of each species was allowed to vary. For the 10 ns window (f) the shorter time constant was fixed to the IRF of window, 200 ps

Section S9 Theoretical Ising model for spatially varying CD signal

In experimentally investigating the CD signal via a spatial probe of the PTPO thin films, we observe two peaks of opposite sign for the anisotropy. We understand this observation as resulting from the fact that, while a given enantiomer of PTPO has a preference for orienting on one side due to interactions of its chiral side chain, it is possible for a domain to be oriented in the less favorable direction. Here, the core distinction is between “face-up” and “face-down” orientations of the conjugated rings as ACD is invariant with respect to in-plane (*xy*) rotation of a given domain.

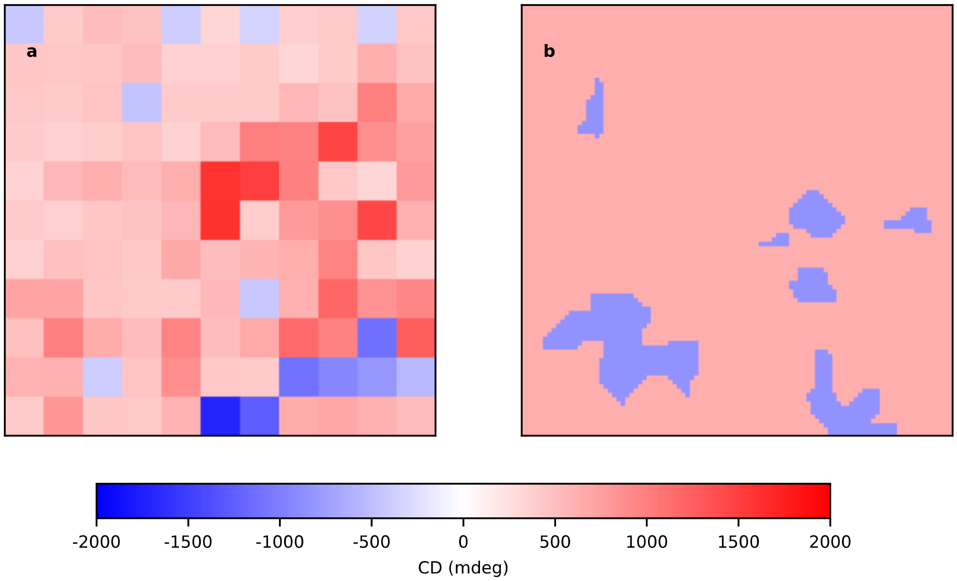

Figure S12. Comparison of spatial dependence of anisotropy for experimental (a) and Ising model (b). For the Ising model, the core parameters are a substrate binding affinity of 16 eV/mm^2 for the positive orientation, a coupling constant of 8 eV/mm making akin adjacent domains favorable, and a temperature quenching from the infinite limit to a thermodynamic beta of 0.05 inverse eV. The length and area-dependent parameters are weighted, respectively, by the discrete areas and lengths of the areas between simulated domains. The domains are constructed as 100 randomized Voronoi regions on a 100 by 100 square pixel gid that simulates a 5 mm by 5mm region. For the two colorings in (b), the values are set by the weighted averages of the positive and negative regions of Figure 4c (-850 mdeg for the negative region, 640 mdeg for the positive one).

To characterize the two different peaks, we implement a modified Ising model[27] in Python 3.10 (numpy (1.22.3) [2], scipy (1.7.3) [3]) of the crystal domains. To mimic the differently shaped domains, we first constructed a Voronoi-Delaunay tessellation [28] of a finite 2D plane wherein a number of domains are formed from the points in a 2D lattice with the lowest Manhattan (taxicab) distance [29] to randomly-chosen points. This simple method mimics different points of crystallization and produces domains of shapes and sizes nearly identical to that of scanning electron microscopy of thiophene thin films, which are triclinic crystals [4].

The modified Ising Hamiltonian reads:

$$H = -\sum_{i,j} J_{ij}\sigma_i\sigma_j - \sum_i h_i\sigma_i$$

where σ_i is the sign of each domain (± 1 for face up/down), J_{ij} is an intersite coupling term weighted by the length of the edges between domains on the lattice to mimic van der Waals forces, and h_i is a term weighted by domain area to account for substrate affinity for one domain orientation over the other.

Here, domains of the same orientation are assumed to have a favorable energetic interaction while the substrate exhibits a modest bias for one orientation. Beginning from a random system depicting an infinitely high temperature, the Ising system is quenched to a finite temperature and propagated according to a Metropolis-Hastings algorithm[30,31] for an arbitrarily long time such that the net degree of anisotropy has converged to a value similar to that of the experimental spatial maps. In contrast with experiment, the Ising model here only permits two orientations and corresponding CD values as opposed to the wider peaks of the experimental histogram (Figure 3, main text). A comparison of the spatial maps of the maximum CD value in the region 420-520 nm is shown below (Figure S11).

One probable source for the experimental dispersion of CD values and existence of thin regions of high magnitude is that of enhanced ACD at interfaces between different domains [32]. Properly modelling such interactions requires both more information on the crystals than is presently known and a full modelling of the 3-dimensional cavity system, which are both beyond the scope of this paper.

Section S10 Comparison with planar chirality in microcavity as demonstrated by Gautier et al [33]

Previous work [33] has demonstrated the efficacy of using the planar chirality of strained films to produce net chiroptical responses in optical microcavities, albeit at oblique incidence. In terms of the Mueller CD term given in terms of transmittances as ½ ln $\left(\frac{T_R}{T_L}\right)$ and in the limit where ellipticity and circular dichroism may be linearly interrelated the conversion to CD (mdeg) is via a factor of $32980 \cdot \frac{2}{\ln(10)}$. For the observed maximal CD in Figure 2a of Reference [33] , the maximum of .02 CD (Mueller) becomes a maximum CD (mdeg) of 600 mdeg.

Section S11 Comparison of home-built and laboratory CD instrument

The behavior of samples measured with the home-built spectrometer also tracks with the values acquired using a commercial CD spectrometer on the same HR substrate, as shown in Figure S12.

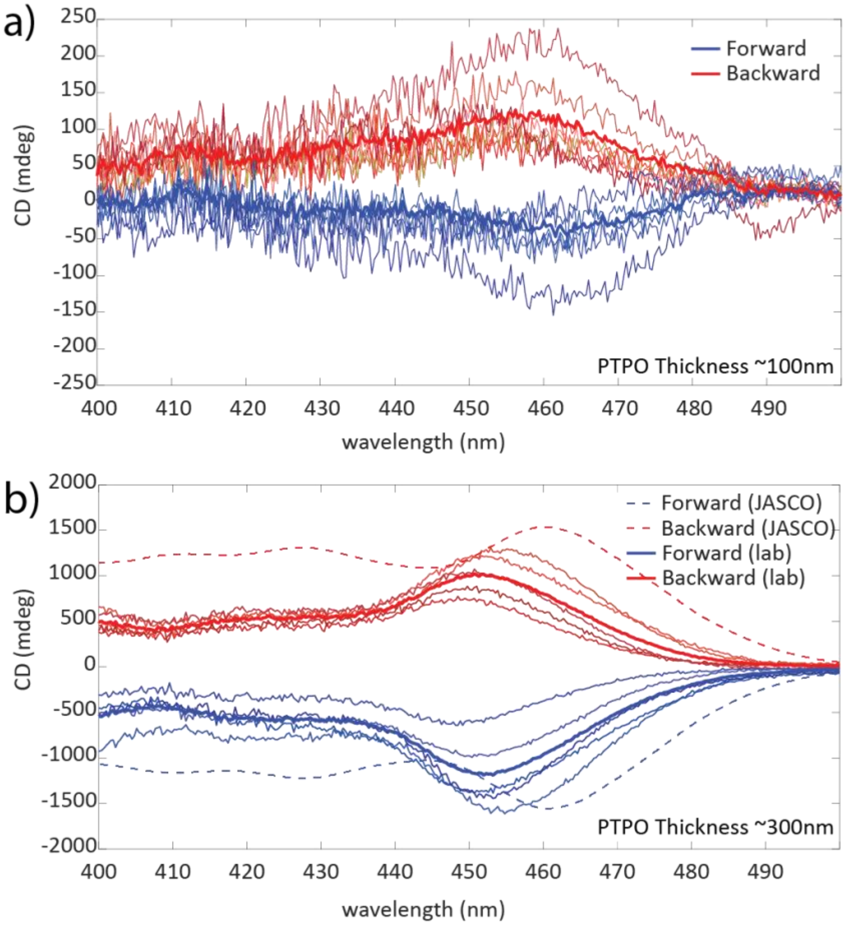

Figure S13. Chiroptical properties of 100 nm (a) and 300 nm (b) PTPO film on an HR substrate measured at different spatial locations. Blue curves are acquired in the forward geometry and red curves are acquired in the backwards geometry. The thick solid curves show the average from several different spots in the backward or forward direction. Plots in a) were obtained using a homebuilt CD spectrometer. Plots labeled "lab" in b) were also acquired on the homebuilt spectrometer. Plot b) also includes characterization of the same film with a commercial spectrometer (JASCO-1500), which shows qualitative agreement with laboratory-measured samples.

Section S12 Cavity Field Visualization

Using the transfer matrix method, one can back-propagate waves to obtain a field visualization[34] in the plane of incidence. In doing so, we obtained visualizations of the cavity modes, with in-plane intensities shown below in Figure S13.

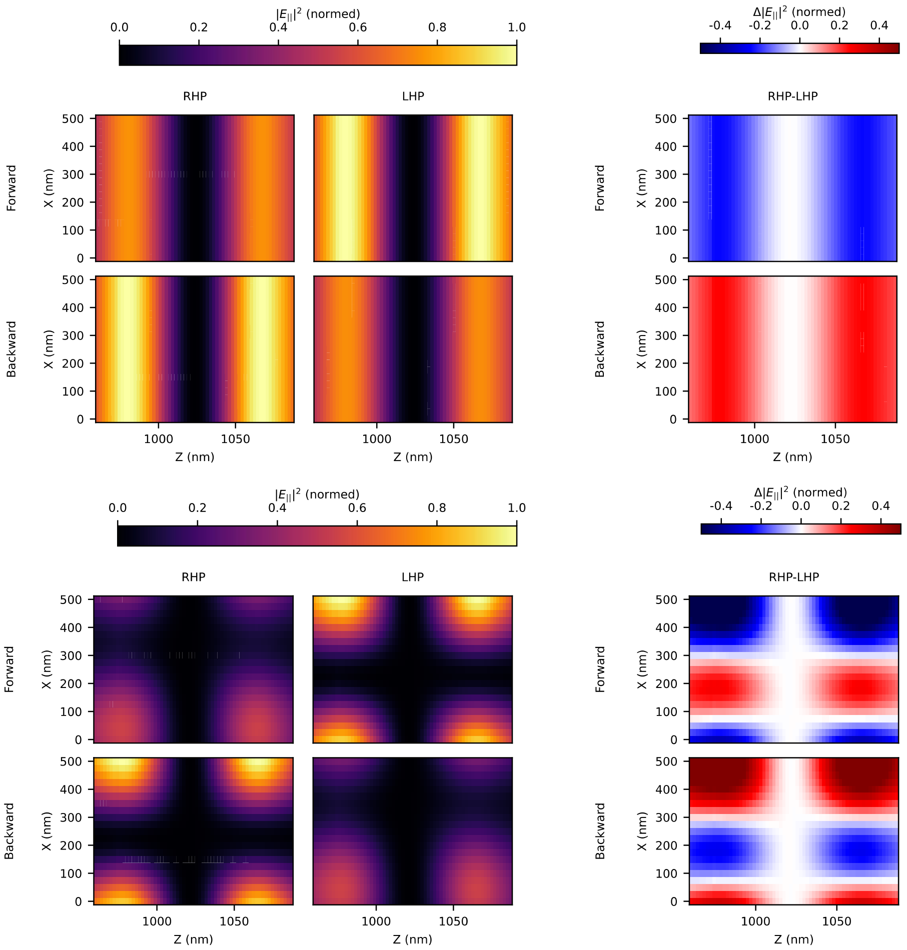

Figure S14. Theoretical in-plane (*xy*) field intensity distributions for PTPO cavity illuminated at resonant wavelength 440 nm at normal incidence (top set) and an angle of incidence of 21 degrees in the *xz* plane. SiO_2 spacer included to tune theoretical cavity resonance to that of experimental cavity, mimicking the PVA spacer. Field intensities normalized to maximum of right-handed (RHP) and left-handed (LHP) illuminations. DBR field distributions omitted.

Section S13 Definitions of Circular Dichroism

There exist competing definitions of the term "circular dichroism" (CD) amongst different scientific communities. One standard definition of CD denotes the differential absorption of LCP and RCP by a sample and may be reported in units of absorbance or ellipticity. These units are regularly interconverted, and such signals are frequently reported as relative measurements (i.e., g-factor).[35-37] This definition of CD considers primarily the observed signal and does not necessarily account for the differential contributions to the net signal via intrinsic and extrinsic 3D and 2D chiral effects. However, the chemistry community is beginning to address observed CD with additional detail as more is learned about the contribution of anisotropic effects to chiroptical signals.[38]

The metamaterials community distinguishes between 3D and 2D chirality through the complex circular transmission matrix, which connects the transmitted field vector and the incident field

vector.[39] Under this definition, the difference in LCP and RCP transmission intensity in the case of CD arises from an asymmetry between the co-polarized t_{ll} and t_{rr} elements; this measure of 3D chirality is independent of the direction of light propagation. In "conversion circular dichroism" (CCD), however, the difference in LCP and RCP transmission intensity results from an asymmetry between the cross-polarized t_{lr} and t_{rl} elements; this measure of 2D chirality indicates partial conversion of the polarization state of the wave and inverts light travelling in the opposite *z* direction.[40,41]

In this communication, we have chosen to utilize the former definition of circular dichroism focusing on a simple differential absorption of LCP and RCP. This is due in part to the heterogeneity of our samples (which would complicate the quantitative determination of the complex circular transmission matrix) as well as the specifics of our experimental design, which allowed only for the relative comparison between transmitted intensities of LCP and RCP and not the differential absorption which would have required both the incident and transmitted intensities of LCP and RCP.

The derivation of Equation 1 is as follows from Reference 36, beginning with the commonly accepted definition of CD in the chemistry community,

$$\mathrm{CD}_{absorbance} = \Delta\mathrm{A} = \mathrm{A}_{\mathrm{LCP}} - \mathrm{A}_{\mathrm{RCP}},$$

where A_i refers to the absorbance of a particular polarization. The absorbance,

$$\text{Absorbance} = -\log_{10}\left(\frac{\mathrm{I}}{\mathrm{I}_0}\right) = \log_{10}(\mathrm{I}_0) - \log_{10}(\mathrm{I})$$

can be related to the logarithm base ten of the ratio of incident intensity (I_0) to transmitted intensity (I). Thus, CD can be recast as the log base ten of the ratio of transmitted intensities, assuming that I_0 is the same for both RCP and LCP, as experimentally verified.

$$\begin{aligned}\mathrm{CD}_{absorbance} &= \log_{10}\left(\frac{\mathrm{I}_0}{\mathrm{I}_{\mathrm{LCP}}}\right) - \log_{10}\left(\frac{\mathrm{I}_0}{\mathrm{I}_{\mathrm{RCP}}}\right) \\ &= \log_{10}(\mathrm{I}_0) - \log_{10}(\mathrm{I}_{\mathrm{LCP}}) - \log_{10}(\mathrm{I}_0) + \log_{10}(\mathrm{I}_{\mathrm{RCP}}) \\ &= \log_{10}(\mathrm{I}_{\mathrm{RCP}}) - \log_{10}(\mathrm{I}_{\mathrm{LCP}}) \\ &= \log_{10}\left(\frac{\mathrm{I}_{\mathrm{RCP}}}{\mathrm{I}_{\mathrm{LCP}}}\right)\end{aligned}$$

Finally, CD is related to ellipticity and expressed in millidegrees, a commonly used unit,

$$\mathrm{CD}_{millidegrees} = 32980 \times \mathrm{CD}_{absorbance}$$

The factor of 32980 is approximately equal to,

$$= 1000\frac{\ln(10)}{4}\frac{180}{\pi}$$

and accounts for the shift from degrees to millidegrees, the shift from base 10 to base e logarithms, and includes additional factors of 2 for an intensity to electric field conversion and from a Taylor expansion.

Supplementary References:

1. Hunter, J. D. Matplotlib: A 2D Graphics Environment. *Comput. Sci. Eng.* **9**, 90–95 (2007).
2. Harris, C. R. *et al.* Array programming with NumPy. *Nature* **585**, 357–362 (2020).
3. Virtanen, P. *et al.* SciPy 1.0: fundamental algorithms for scientific computing in Python. *Nat Methods* **17**, 261–272 (2020).
4. Zinna, F. *et al.* Emergent Nonreciprocal Circularly Polarized Emission from an Organic Thin Film. *Adv. Mater.* **32**, 2002575 (2020).
5. Lee, H. Y., Peng, Y. & Shkel, Y. M. Strain-dielectric response of dielectrics as foundation for electrostriction stresses. *Journal of Applied Physics* **98**, 074104 (2005).
6. Shkel, Y. M. & Klingenberg, D. J. Material parameters for electrostriction. *Journal of Applied Physics* **80**, 4566–4572 (1996).
7. Atanackovic, T. M. & Guran, A. *Theory of Elasticity for Scientists and Engineers*. (Springer Science & Business Media, 2000).
8. Çetinörgü, E. A new method to experimentally determine the thermal expansion coefficient, Poisson's ratio and Young's modulus of thin films. *J Mater Sci* **44**, 2167–2170 (2009).
9. Shkel, Y. M. & Klingenberg, D. J. Electrostriction of polarizable materials: Comparison of models with experimental data. *J. Appl. Phys.* **83**, (1998).
10. Salij, A., Goldsmith, R. H. & Tempelaar, R. Theory of Apparent Circular Dichroism Reveals the Origin of Inverted and Noninverted Chiroptical Response under Sample Flipping. *J. Am. Chem. Soc.* **143**, 21519–21531 (2021).
11. Shao, Y. *et al.* Advances in molecular quantum chemistry contained in the Q-Chem 4 program package. *Molecular Physics* **113**, 184–215 (2015).

12. Frisch, M. J., Pople, J. A. & Binkley, J. S. Self‐consistent molecular orbital methods 25. Supplementary functions for Gaussian basis sets. *The Journal of Chemical Physics* **80**, 3265–3269 (1984).
13. Chai, J.-D. & Head-Gordon, M. Long-range corrected hybrid density functionals with damped atom–atom dispersion corrections. *Phys. Chem. Chem. Phys.* **10**, 6615 (2008).
14. Vérité, P. M., Guido, C. A. & Jacquemin, D. First-principles investigation of the double ESIPT process in a thiophene-based dye. *Phys. Chem. Chem. Phys.* **21**, 2307–2317 (2019).
15. Yanai, T., Tew, D. P. & Handy, N. C. A new hybrid exchange–correlation functional using the Coulomb-attenuating method (CAM-B3LYP). *Chemical Physics Letters* **393**, 51–57 (2004).
16. Hellweg, A. & Rappoport, D. Development of new auxiliary basis functions of the Karlsruhe segmented contracted basis sets including diffuse basis functions (def2-SVPD, def2-TZVPPD, and def2-QVPPD) for RI-MP2 and RI-CC calculations. *Phys. Chem. Chem. Phys.* **17**, 1010–1017 (2015).
17. Tomasi, J., Mennucci, B. & Cancès, E. The IEF version of the PCM solvation method: an overview of a new method addressed to study molecular solutes at the QM ab initio level. *Journal of Molecular Structure: THEOCHEM* **464**, 211–226 (1999).
18. Bay, M. M., Vignolini, S. & Vynck, K. PyLlama: A stable and versatile Python toolkit for the electromagnetic modelling of multilayered anisotropic media. *Computer Physics Communications* **273**, 108256 (2022).
19. Ko, D. Y. K. & Sambles, J. R. Scattering matrix method for propagation of radiation in stratified media: attenuated total reflection studies of liquid crystals. *J. Opt. Soc. Am. A* **5**, 1863 (1988).

20. Berreman, D. W. Optics in Stratified and Anisotropic Media: 4x 4-Matrix Formulation. *Journal of the Optical Society of America* **62**, 502–510 (1972).

21. Yeh, P. *Optical Waves in Layered Media*. (John Wiley & Sons Inc., 1988).

22. Redheffer, R. Difference equations and functional equations in transmission-lines theory. in *Modern Mathematics for the Engineer* 282–337 (McGraw-Hill, 1961).

23. Rumpf, R. C. Improved Formulation of Scattering Matrices for Semi-analytical Methods that is Consistent with Convention. *PIER B* **35**, 241–261 (2011).

24. Agranovich, V. M. & Gartstein, Yu. N. Spatial dispersion and negative refraction of light. *Uspekhi Fizicheskikh Nauk* **176**, 1051 (2006).

25. Gil, J. J. & Ossikovski, R. *Polarized Light and the Mueller Matrix Approach* (2nd Ed.). (CRC Press, 2022).

26. Kuroda, R., Harada, T. & Shindo, Y. A solid-state dedicated circular dichroism spectrophotometer: Development and application. *Review of Scientific Instruments* **72**, 3802–3810 (2001).

27. Ising, E. Beitrag zur Theorie des Ferromagnetismus. *Zeitschrift für Physik* **31**, 253–258 (1925).

28. Lima, F. W. S., Moreira, J. E., Andrade, J. S. & Costa, U. M. S. The ferromagnetic Ising model on a Voronoi–Delaunay lattice. *Physica A: Statistical Mechanics and its Applications* **283**, 100–106 (2000).

29. Okabe, A., Boots, B. & Sugihara, K. Nearest neighbourhood operations with generalized Voronoi diagrams: a review. *International Journal of Geographical Information Systems* **8**, 43–71 (1994).

30. Metropolis, N. & Ulam, S. The Monte Carlo Method. *Journal of the American Statistical Association* **44**, 335–341 (1949).

31. Hastings, W. K. Monte Carlo sampling methods using Markov chains and their applications. *Biometrika* **57**, 97–109 (1970).

32. Catalano, L. *et al.* Sequencing and Welding of Molecular Single-Crystal Optical Waveguides. *Adv. Funct. Mater.* **30**, 2003443 (2020).

33. Gautier, J., Li, M., Ebbesen, T. W. & Genet, C. Planar Chirality and Optical Spin–Orbit Coupling for Chiral Fabry–Perot Cavities. *ACS Photonics* **9**, 778–783 (2022).

34. Passler, N. C. & Paarmann, A. Generalized 4 × 4 matrix formalism for light propagation in anisotropic stratified media: study of surface phonon polaritons in polar dielectric heterostructures. *J. Opt. Soc. Am. B* **34**, 2128–2139 (2017).

35. Barron, L. D. *Molecular Light Scattering and Optical Activity* (2nd Ed.). (Cambridge University Press: Cambridge, 2004).

36. Rodger, A.; Nordén, B. *Circular Dichroism and Linear Dichroism*. (Oxford University Press, 1997).

37, Berova, N.; Bari, L. D.; Pescitelli, G. Application of Electronic Circular Dichroism in Configurational and Conformational Analysis of Organic Compounds. *Chem. Soc. Rev.* **36**, 914-931 (2007).

38. Ugras, T. J.; Yao, Y.; Robinson, R. D. Can We Still Measure Circular Dichroism with Circular Dichroism Spectrometers: The Dangers of Anisotropic Artifacts. *Chirality* **35**, 846–855 (2023).

39. Cao, T.; Li, Y.; Zhang, X.; Zou, Y. Theoretical Study of Tunable Chirality from Graphene Integrated Achiral Metasurfaces. *Photon. Res.* **5**, 441-449 (2017).

40. Plum, E.; Liu, X.-X.; Fedotov, V. A.; Chen, Y.; Tsai, D. P.; Zheludev, N. I. Metamaterials: Optical Activity without Chirality. *Phys. Rev. Lett.* **102**, 113902 (2009).

41. Menzel, C.; Rockstuhl, C.; Lederer, F. Advanced Jones Calculus for the Classification of Periodic Metamaterials. *Phys. Rev. A* **82**, 053811 (2010).
